# Musculoskeletal, Functional and Performance Impairment in Female Overhead Athletes with a Previous Shoulder Injury

**DOI:** 10.3390/healthcare12010021

**Published:** 2023-12-21

**Authors:** María Belén Alonso-Muñoz, Andrés Calvache-Mateo, Javier Martín-Núñez, Laura López-López, Alba Navas-Otero, Alejandro Heredia-Ciuró, Marie Carmen Valenza

**Affiliations:** Department of Physiotherapy, Faculty of Health Sciences, University of Granada, Av. De la Ilustración, 60, 18071 Granada, Spain; lll92hs@correo.ugr.es (M.B.A.-M.); andrescalvache@ugr.es (A.C.-M.); javimn@ugr.es (J.M.-N.); e.albanavas@go.ugr.es (A.N.-O.); ahc@ugr.es (A.H.-C.); cvalenza@ugr.es (M.C.V.)

**Keywords:** shoulder pain, muscle strength, functionality, upper limbs

## Abstract

Background: Shoulder injuries are substantial problems in overhead athletes, and more studies are necessary to deepen the knowledge on this type of injury. The objective of this study was to compare the overall function and performance of female overhead athletes with and without a previous history of shoulder injuries. Methods: In this cross-sectional study, female overhead athletes with and without a previous shoulder injury were included. Muscular impairment, the stability of the shoulder, strength, scapular dyskinesia, functionality and sports performance were evaluated. A total of 50 females were included. Results: There were significant differences in strength (*p* = 0.046) and stability (*p* = 0.039) between groups, with a poorer score in the group with a history of shoulder injury. Regarding scapular dyskinesia, significant differences were also observed between groups (*p* = 0.048), with higher levels of dyskinesia in the group with previous shoulder injury. Also, muscular impairment showed significant differences between groups for the three muscles evaluated (*p* < 0.005). Additionally, the group without a previous shoulder injury presented with a significantly greater score in functionality (*p* = 0.046) and sports performance (*p* = 0.004). Conclusion: In conclusion, previous shoulder injuries are an important factor to take into account in female overhead athletes. Players with a history of shoulder injury present clinical impairments during the game, leading to poorer functional status and performance in sport.

## 1. Introduction

Sport-related injuries account for one-fifth of injury-related visits to emergency departments by patients aged 5–24 years [1]. Most studies in the literature are concerned with analyzing the prevalence or incidence of diagnosed injuries in sport [2]. However, few studies have addressed the recovery of sport-related injuries and recurrences.

Shoulder injuries and shoulder pain are substantial problems in overhead athletes, such as baseball and softball pitchers or handball, volleyball, cricket and tennis players [3]. Most of the reported shoulder injuries are related to strains that have caused a degenerative process leading to injury [4]. During overhead sports play, the shoulder girdle can become overloaded and stressed due to collisions, falls, and overhead throws combined with high-velocity movements of the upper limb [5].

The shoulder is a problematic area, due to its repetitive, demanding movement pattern that can sometimes lead to long-term, or even permanent, damage to the affected structures, associated with joint vulnerability [5] and injury rates [6]. However, there are sex and age differences in shoulder injury patients, and other injuries which have been observed in several other anatomical sites and different sports [7]. Biomechanical factors, such as shoulder mobility, coordination and impaired throwing technique, may also be risk factors for shoulder injuries in overhead sports [8,9]. Other factors such as fatigue have been suggested to possibly affect the shoulder’s strength, proprioception and range of motion, therefore representing possible risk factors for overuse shoulder injury [10].

Rotator cuff alterations without symptoms are frequently observed in athletes involved in overhead activities and throwing sports [11]. In addition, changes in the shoulder stabilizing muscles, evidenced by decreased strength in the predominant medial trapezius and an imbalance in glenohumeral rotator muscle strength, are related to a past experience of shoulder discomfort in overhead athletes [12]. All of this may increase the risk of functional mobility disorder [13], muscular weakness [14] and instability [5] that can contribute to generating and perpetuating harmful patterns that can lead to recurrences [4]. Conservative shoulder injury treatment has been shown to be less effective than in other joints [15,16]. The main limitations of the treatment and recovery of these injuries are the difficulties in identifying the injured functions and motor structures [5].

The topic of shoulder pain and injury in overhead athletes has been widely researched and described for years [17,18,19,20]. Given the importance and prevalence of shoulder injuries in this population, it is important to develop a shoulder assessment that allows for anticipating injuries. Numerous studies have conducted assessments using highly precise instruments such as electromyography [21,22], isokinetic [23] or sonographic evaluation [24].

However, despite the high reliability of these instruments, translating these assessments into clinical practice can be challenging.

Therefore, the main objective of this study was to compare the overall function and performance of female overhead athletes with and without a previous history of shoulder injuries using easily reproducible tests in the clinical setting. 

## 2. Methods

### 2.1. Study Design

A cross-sectional study was conducted between September and November 2020. This observational study sought to examine the clinical impairments presented in female overhead athletes with a previous history of shoulder injuries, in addition to comparing the overall function and performance of these athletes with healthy controls. The study was approved by a local committee on research ethics.

### 2.2. Setting 

The research was conducted in “Facultad de Ciencias de la Salud” (Granada). The medical facilities allowed the assessment of athletes, including representative tools for the performance of these assessments in any clinical setting.

### 2.3. Participants

The sample of the study was composed of female overhead athletes. The inclusion criteria were (i) being female; (ii) being a female overhead athlete; (iii) being actively involved in playing overhead sport for at least 8 h/week; (iv) being 18 years old or more; and (v) having experienced a previous shoulder injury such as muscle strain, muscle tear, tendonitis. The exclusion criterion was (i) any joint injury that did not allow sports participation or the individual to perform the study tests. 

Healthy controls with the following inclusion criteria were included: (i) being female; (ii) being a female overhead athletes; (iii) being actively involved in playing overhead sport for at least 8 h/week; (iv) being 18 years old or more; and (v) not having a previous shoulder injury.

The exclusion criteria were (i) a prior history of surgery on the shoulder or neck; (ii) fractures in the shoulder region; (iii) experiencing symptoms in both shoulders simultaneously; (iv) cervical radiculopathy; (v) prior interventions involving steroid injections; or (vi) any form of physical intervention in the neck-shoulder area within the preceding year.

This study was performed in accordance with the Declaration of Helsinki [25]. All candidates were informed about the study conditions and signed the informed consent form, and after verifying the absence of exclusion criteria, they were included. The sample was recruited from different female overhead sports teams (e.g., volleyball, basketball, handball). 

### 2.4. Procedures

All participants were categorized into two groups based on the presence of a history of shoulder injury. In addition, they were submitted to a comprehensive questionnaire regarding anthropometric and clinical variables, including factors such as age, dominant side, and history of shoulder injury at baseline. 

Concurrently, data were collected on musculoskeletal injury variables, including assessment of muscle impairment, shoulder stability, strength, and scapular dyskinesia. In addition, an assessment of functionality and sports performance was performed.

### 2.5. Variables Measures

The main variables were muscular impairment, stability of the shoulder, strength, scapular dyskinesia, functionality and sports performance. All the tests were carried out in the injured shoulder.

#### 2.5.1. Muscular Impairment

To assess muscular impairment, muscle trigger points (TrPs) were explored in the infraspinatus, supraspinatus and subscapularis muscles due to their prevalence in athletes with shoulder injury [26]. TrPs diagnosis was performed following the criteria described by Simons et al.: (1) palpable taut band in a skeletal muscle; (2) hypersensitive spot in the taut band; (3) local twitch response elicited by the snapping palpation of the taut band; and (4) presence of referred pain in response to compression [27]. When implemented by a skilled evaluator, these criteria have demonstrated a favorable inter-examiner reliability (k) in the range of 0.84 to 0.88, as reported by Gerwin et al. [28]. All TrPs were assessed by an evaluator with over 5 years of experience in diagnosing muscular myofascial pain syndrome, and who was blinded to the subjects’ condition.

Active myofascial trigger points (PG) were identified when stimulation induced both local and referred pain, replicating the subject’s known pain symptoms, and when the subject could recognize the pain as familiar. Conversely, latent TrPs were determined when compression-induced local and referred pain failed to reproduce any recognizable symptoms [27]. After assessing each muscle’s TrPs, participants were asked to report whether the palpation-induced discomfort in both the specific area and other regions (referred pain) resembled any symptoms they usually experience. This information helped to determine if the pain elicited during palpation replicated familiar symptoms or triggered different and unfamiliar sensations.

#### 2.5.2. Stability of the Shoulder

The Y Balance Test-Upper Quarter (YBT-UQ) was performed to assess the stability of the shoulder. The participants had to reach in the medial, superolateral, and inferolateral directions to the furthest possible point they could. For an analysis of overall performance, the composite reach distance was calculated by averaging the greatest trial in each of the 3 normalized reach distances [29].

For this test, a posture platform was used, to which three pieces of tubing were attached in the medial, inferolateral and superolateral directions, thus allowing the assessment of shoulder mobility in these directions. By pushing on these pieces, the subject was able to standardize the reach height (how far the hand is from the starting position). In order to make the measurement as accurate as possible, C7 was identified and the distance from the spine to the most distal tip of the right middle finger was measured with the shoulder in 90º abduction, in such a way as to adjust the distance of the extremity with the greatest reach obtained in each of the directions [29].

Due to the difficulty of the test, prior to the test, the evaluator explained the procedure and showed each participant how to perform the test correctly. A rehearsal was performed prior to the test to ensure the correct performance of the test. It should be noted that the test was discarded when the following situations occurred: (i) lifting the foot off the floor; (ii) not being able to return the extended hand to the initial starting position; (iii) using the reach indicator to assist the target posture; (iv) losing contact between the extended hand and the reach indicator (pushing it); and (v) not maintaining the initial unilateral position with the support hand [29]. The tests were evaluated by two blinded evaluators simultaneously to reduce bias. When no agreement was reached, a third researcher conducted the evaluation.

#### 2.5.3. Strength

The Seated Medicine Ball Throw (SMBT) [30] is an open kinetic chain functional screening test to assess muscle strength. The subjects were instructed to throw a 2 kg medicine ball as far as possible. For further analysis, the mean distance of four test trials was calculated [31].

The initial position of the participants was seated with their upper back against the back of the seat and the ball controlled with their hands on their chest waiting for the signal to start the throw. To calculate the throwing distance, the starting point was the front edge of the seat, and the end point was the rearmost contact of the medicine ball at first impact. 

A blinded evaluator instructed the participants by explaining the test and two warm-up throws, after which the participants were given a one-minute break before the four throws were performed. The test should be considered valid when the participant maintained back contact with the back of the chair and the throw was clearly felt as their maximum effort.

#### 2.5.4. Scapular Dyskinesia

Two blinded evaluators performed the scapular movement evaluations of each participant by standing three meters away for observation from the posterior. The patient raised their arms in forward flexion to their maximum elevation and then lowered them to the starting position while holding an external weight of one kilogram. This exercise was performed three to five times. The prominence of any aspect of the medial scapular border was recorded as “yes” (prominence detected) or “no” (prominence not detected). When no agreement was reached, a third researcher conducted the evaluation.

The clinical assessment of medial border prominence in symptomatic patients has shown a correlation with biomechanically identified dyskinesis. This approach is sufficiently reliable to serve as the foundation for establishing the presence or absence of dyskinesis [32,33,34].

#### 2.5.5. Functionality 

The Disabilities of the Arm, Shoulder, and Hand (DASH) is a self-administered region-specific outcome instrument developed as a measure of self-rated upper-extremity symptoms and disability and has been identified as the most validated and easy to use measure of upper extremity function [35]. The DASH consists of 30 core items (each item is scored on a 5-point scale) which generate a disability score, scaled 0 (no disability) to 100 (severe disability) [36].

The effectiveness of the DASH questionnaire within the context of upper extremity orthopedic trauma is substantiated by its status as the extensively validated tool for assessing upper extremity function in disorders affecting this anatomical region [37].

The DASH questionnaire presents a high internal reliability (Cronbach’s Alpha of 0.96), as well as a high construct validity when compared to the dimensions of the SF-36 (ranged between 0.36 to 0.62) [38].

#### 2.5.6. Sports Performance

The Kerlan–Jobe Orthopaedic Clinic (KJOC) questionnaire was developed to assess overhead athletes’ functional status and responsiveness to interventions. It is sensitive to upper extremity throwing dysfunction. The KJOC is a scale focusing on the functional and performance parameters, interpersonal relationships and symptoms of overhead athletes [39].

The ten-item KJOC score is composed of two sections, each of which contains five questions that probe into athletes’ perceptions of their shoulder and elbow function and performance. Participants mark their responses on a ten-centimeter Visual Analog Scale (VAS), with the left margin representing zero points and the right margin indicating ten points. A higher score indicates better upper arm function. Item scores are measured with a ruler from the left corner of the scale to the point marked by the participant, and recorded in centimeters to one decimal place. The overall score is the total of all items, resulting in a score between 0 and 100 points, where 100 points means optimal upper extremity function [40].

### 2.6. Statical Analysis

The data obtained in the study were analyzed exhaustively with the Statistical Package for the Social Sciences (SPSS) version 20.0. The study participants were classified into two distinct groups: one group consisted of individuals with a documented history of shoulder injuries, while the other group constituted the control group, consisting of individuals with no history of such injuries.

To provide a detailed view of the characteristics of the sample, quantitative variables were expressed as mean values accompanied by their respective standard deviations. Before proceeding with the analysis, the normality of the data was assessed using the Kolmogorov-Smirnov (K-S) test. The examination of associations between categorical variables was carried out using Pearson’s χ^2^ test.

For examination of the relationship between a dichotomous independent variable and a quantitative dependent variable with a parametric distribution, the Student’s test for independent samples was used. In cases where the assumption of normality was not met, as indicated by the Kolmogorov–Smirnov test, the Mann–Whitney U test was applied. The measure of the effect was quantified in terms of differences between medians. In all cases, a significance level of *p* < 0.05 was established as the threshold for statistical significance.

## 3. Results

**Figure 1:**
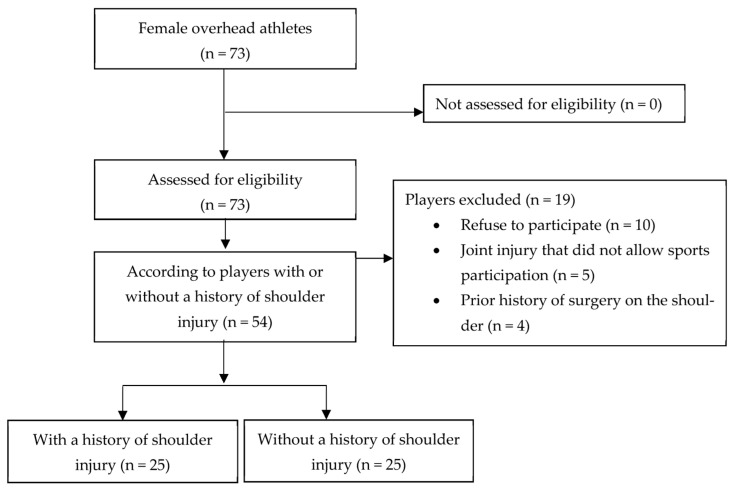
The STROBE flowchart.

Our study included 50 female overhead athletes, 25 with a previous shoulder injury and 25 without a previous shoulder injury (Figure 1).

The characteristics of the study population are summarized in Table 1. 

As we can see in Table 1 the mean age was similar in both groups, being 20.4 ± 1.9 years in the group of players with a history of shoulder injury and 18.8 ± 1.2 in those players who had no history of shoulder injury. For both groups, their right was their dominant side (83.9% vs. 88.9%). There were no significant differences between groups according to the body mass index (*p* > 0.05), and participants in both groups were normal weight (BMI ranging between 18.5 to 24.9). In addition, the most prevalent sport among the participants was handball. All the participants with a history of shoulder injury had received physiotherapy treatment consisting of stretching exercises and targeted exercise.

The differences between the groups with regard to the main variables are presented in Table 2.

As shown in Table 2, there were significant differences between groups in the number of TrPs present in the three muscles evaluated: in the supraspinatus (*p* ≤ 0.001), TrPs were present in 24.3% of the players with a previous shoulder injury and were not present in any of the players without a previous shoulder injury; in the subscapularis (*p* ≤ 0.001), TrPs were present in 21.3% of the players with a previous shoulder injury and were not present in any of the players without a previous shoulder injury; and in the infraspinatus (*p* ≤ 0.003), with worse results in the group with a history of shoulder injuries, TrPs were present in 24.3% of the players with a previous shoulder injury and only 11.1% of those players without a previous shoulder injury.

Additionally, there were significant differences in strength (*p* = 0.046): it was observed that players with no previous shoulder injury (4.1 ± 1.7) showed more strength than those with a history of shoulder injury (3.1 ± 0.5), and therefore, the latter threw the medicine ball less far. In relation to stability, differences were also found in favor of the group without a previous shoulder injury (*p* = 0.039), with a poorer score in the group with a history of shoulder injury (52.3 ± 8.9). Regarding scapular dyskinesia, significant differences were also observed between groups (*p* = 0.048), with higher levels of dyskinesia in the group with a history of shoulder injury (66.7%).

Therefore, the group without a history of shoulder injury presented with a significantly greater score in DASH (*p* = 0.046). Similarly, when sporting performance was assessed using the KJOC questionnaire, a significant difference (*p* = 0.004) was observed in favour of the group without a previous shoulder injury, which presented with higher sporting performance (91.6 ± 7.8) than those players who had a history of injury to this joint (85.3 ± 8.4). 

## 4. Discussion

The aim of this study was to describe the clinical impairments present in female overhead athletes with a previous history of shoulder injuries and to compare the overall function and performance of these players with healthy controls. The included sample in this study was similar to others reported in published studies [41], and this allowed us to compare our results with others.

In this study, only female overhead athletes were included. Sex-based differences have been reported in morphological and neuromuscular characteristics of such muscles [42] and might be associated with different adaptations to training and clinical treatments [43]. One example is the study carried out by Deodato et al. (2023), which found sex-based differences in abdominal and lumbar muscles characteristics in adolescent gymnasts [44].

For the last few years, different studies have been focused on a wide variety of competitive athletes with shoulder pain [45,46]. This is due to its prevalence and functional repercussions. There is a growing interest in the different clinical aspects affecting the performance of this population, which is due to the low effectiveness of the therapeutic options. These aspects have become more important, since studies focusing on different sports like volleyball [47], softball [48], baseball [49], tennis [50] and swimmers [51] players have shown poorer function and performance after recovery from a shoulder injury. However, to the best of our knowledge, this is the first attempt to analyze the shoulder-related musculoskeletal and clinical impairments in female overhead athletes after a shoulder injury.

Our results show an important impact of previous shoulder injuries on muscular impairment, with a greater presence of TrPs in this group of patients for the supraspinatus, subscapularis and infraspinatus muscles. Bailón-Cerezo et al. [52] and Hidalgo-Lozano et al. [53] presented similar results analyzing a sample of competitive swimmers which showed a greater presence of TrPs in swimmers with shoulder pain compared to swimmers without. 

In our study, our results demonstrate that players with a previous shoulder injury presented worse stability of the affected limb, limiting the possible performance of the shoulder. Several studies evaluating the stability of the shoulder in competitive athletes have used the YBT-UQ test, finding it appropriate and easy to use [54,55,56,57]. Beyranvand et al. [54] and Zandi et al. [56] found significantly lower YBT-UQ scores in all directions in athletes with ongoing shoulder pathologies, similar to our results. In contrast, Kim et al. [49] found that there were no group differences in the medial and inferolateral directions of the YBT-UQ, but the superolateral direction showed lower mean scores in the injured shoulder group versus the uninjured group. The main reason for the difference between the study of Kim et al. [49] and our results could be the different profile of lesions included in Kim et al.’s study. Our population have previously resolved lesions related to the shoulder girdle.

Our results also found poorer performance in muscle strength in the group with previous shoulder injuries when compared to the group without. Contrary to the results of our study, the study of Forthomme et al. [14] did not find any muscle strength differences between handball players with and without a previous history of injury. These differences between results could be due to the use of isokinetic analysis of muscle strength, and the use in our case of a more global strength test. Borms et al. [31] reported that SMBT is a reliable alternative for evaluating upper extremity strength. 

The results obtained on scapular dyskinesia showed that the group with previous injury presented significantly higher levels of dyskinesia when compared to the group without previous injury. Although scapular dyskinesia is common among overhead athletes with shoulder pain [57], it has also been demonstrated to be common among asymptomatic athletes [58]. Clarsen et al. [7] found results similar to our study and showed the association between scapular dyskinesis and shoulder injury. 

In our study, players with previous shoulder injury presented poorer upper-extremity symptoms and disability. De Oliveira et al. [45] showed that overhead athletes with shoulder pain had lower values for the DASH questionnaire. Moreover, shoulder pain was one of the most frequently reported complaints among handball athletes, even when they were not training. Asker et al. [33] found, in a study with elite handball players, that female players had an increased shoulder injury rate due to a weakness in the shoulder rotations during the pre-season.

Our results also found poorer functional status and physical performance of players with a history of shoulder injury during the game. Sciascia et al. [59] found similar results in 738 collegiate athletes and showed that athletes with a previous shoulder injury had a lower perceived physical capacity. Merkel [60] showed that dysfunction, pain chronicity, the increase in sport withdrawal and injury recurrence might be the result of a rapid return to sport without an adequate rehabilitation. 

Among the limitations of our study, we should highlight that the group with a previous shoulder injury were not analyzed for the etiology of their injury. Another important limitation was the number of subjects included in the current study. Moreover, shoulder strength measurement was limited to SMBT, despite the most reliable shoulder strength measures being isometric contraction in different planes and degrees using a handheld dynamometer and eccentric function using an isokinetic dynamometer [49]. Another limitation is that the phase of the menstrual cycle was not recorded, and this could be a factor that affects pain modulation. 

The strength of our study lies in the fact that the tools used are easily reproducible in clinical practice. They do not require expensive instruments but can be employed using affordable materials. All of the tests included have been validated and used in other studies [32,39,44,61].

Finally, the evidence of causality was not claimed, due to the methodological limitations of this study and variable reliability, and the relationship between clinical impairment and previous shoulder injury remains unclear in female overhead athletes. Future longitudinal studies are required to examine the evidence of causality.

The conservative treatment of shoulder injuries presents difficulties in identifying the injured function and motor structures [62]. A biomechanically altered shoulder joint increases the risk of shoulder injuries in female overhead athletes, and previous shoulder injuries can generate harmful patterns during the game, increasing the recurrence of injuries [4]. Nevertheless, our study provided important data to better understand the clinical impairments of previous shoulder injuries and lays a foundation to establish a specific intervention.

Based on the findings presented in this article, future directions in the field of sports medicine and rehabilitation for female overhead athletes with a history of shoulder injuries could focus on targeted interventions to address the identified clinical impairments. This study emphasizes the impact of previous shoulder injuries on stability, strength, scapular dyskinesia, TrPs, and the functionality and sports performance of female overhead athletes with a history of shoulder injuries. Therefore, future research could explore specific rehabilitation or therapies aimed at addressing these impairments.

## 5. Conclusions

In conclusion, previous shoulder injuries are an important factor to take into account in female overhead athletes. Players with a history of shoulder injuries present clinical impairments during the game. Also, they have a poorer functional status and performance in sport.

## Figures and Tables

**Table 1:** Baseline characteristics of the groups.

	With a History of Shoulder Injury (n = 25)	Without a History of Shoulder Injury (n = 25)	*p* Value
Age (years)	20.4 ± 1.9	18.8 ± 1.2	0.061
Dominant side (%)			
Right	83.9	88.9	0.40
Left	16.1	11.1	

Data are expressed as mean ± SD.

**Table 2:** Differences between groups regarding the main variables.

Variables	With a History of Shoulder Injury (n = 25)	Without a History of Shoulder Injury (n = 25)	*p* Value
Active TrPs (%)			
TrPs supraspinatus	24.3	0	≤0.001 **
TrPs subscapularis	21.3	0	≤0.001 **
TrPs infraspinatus	27.3	11.1	≤0.003 *
YBT-UQ (cm)	52.3 ± 8.9	59.8 ± 7.3	0.039 *
SMBT (m)	3.1 ± 0.5	4.1 ± 1.7	0.046 *
Dyskinesia (%)	66.7	44.4	0.048 *
DASH	7.0 ± 3.7	4.7 ± 4.3	0.046 *
KJOC	85.3 ± 8.4	91.6 ± 7.8	0.004 *

TrPs = trigger points; YBT-UQ: Y Balance Test-Upper Quarter; SMBT: Seated Medicine Ball Throw; DASH = Disabilities of the Arm, Shoulder and Hand questionnaire; KJOC = The Kerlan–Jobe Orthopaedic Clinic. Data are expressed as Mean ± SD. * *p* < 0.05, ** *p* < 0.001.

## Data Availability

Data are contained within the article.
